# HIV Infection as an Independent Factor Accelerating Epigenetic Ageing in Men Treated with Integrase Inhibitors: A Case–Control Study

**DOI:** 10.3390/v18020199

**Published:** 2026-02-02

**Authors:** Mateusz Bożejko, Małgorzata Małodobra-Mazur, Andrzej Gnatowski, Monika Ołdakowska, Aleksandra Szymczak, Bartosz Szetela, Hubert Ciepłucha, Aleksander Zińczuk, Brygida Knysz

**Affiliations:** 1 Department of Infectious Diseases, Liver Disease and Acquired Immune Deficiencies, Wroclaw Medical University, Koszarowa 5, 51-149 Wroclaw, Poland; aleksandra.szymczak@umw.edu.pl (A.S.);; 2 Department of Forensic Medicine, Division of Molecular Techniques, Wroclaw Medical University, Skłodowskiej-Curie 52, 50-369 Wroclaw, Poland; 3 Department of Control Systems and Mechatronics, Faculty of Information and Communication Technology, Wroclaw University of Science and Technology, Janiszewskiego 11/17, 50-372 Wroclaw, Poland; 4 1st Infectious Diseases Ward, J. Gromkowski Provincial Specialist Hospital in Wroclaw, Koszarowa 5, 51-149 Wroclaw, Poland

**Keywords:** HIV, ageing, epigenomics

## Abstract

A number of published studies suggest that HIV infection accelerates epigenetic ageing. The main aim of this study was to ascertain if HIV infection is an independent factor leading to DNA hypomethylation and accelerating epigenetic ageing in men successfully treated with integrase inhibitor (INSTI)-based combined antiretroviral therapy (cART). Forty-eight (48) men living with HIV receiving INSTI-based cART and fifty (50) uninfected men in the control group were included. All participants filled out a questionnaire probing into lifestyle factors. Global and site-specific DNA methylation and expression of methyltransferase genes were examined in all participants. As well, all patients underwent basic laboratory blood tests. The results were analysed using statistical and machine learning methods. We found a strong association between HIV infection and global DNA hypomethylation as well as significant association with higher expression of the methyltransferase gene *DNMT1*. However, there was no association with DNA methylation levels of CNOT2, DPP6, FOXG1 and NPTX2 genes or expression levels of *DNMT3a* and *DNMT3b*. The results confirm that in men successfully treated with INSTI-based cART, HIV infection is an independent factor causing global DNA hypomethylation and increased *DNMT1* expression and thus accelerating epigenetic ageing.

## 1. Introduction

Human immunodeficiency virus (HIV) infection remains a major public health concern worldwide. Since the introduction of combined antiretroviral therapy, HIV has become a chronic infection, and the life expectancy of people living with HIV (PLWH) has levelled with the general population [1,2]. However, observational studies show shorter disease-free survival among PLWH [3]. PLWH are at greater risk of cardiovascular, respiratory, liver and kidney diseases, which might be explained in part by premature ageing [4].

DNA methylation is an important marker of biological ageing. It is commonly used as an ageing clock due to its high accuracy [5]. In general, hypomethylation progresses throughout the genome with age [6]. Furthermore, numerous epigenetic clocks have been established and successfully used for biological age determination based on specific profiles of DNA methylation. Accelerated epigenetic ageing has been associated with, among others, cancer, alcohol consumption, infections and cardiorespiratory disorders [7].

In recent years, several studies have been conducted on epigenetic aspects of ageing in PLWH. A recently published systematic review of studies comparing the intensity of epigenetic ageing in PLWH compared to uninfected individuals [8] suggests accelerated epigenetic ageing in PLWH, both those treated with combined antiretroviral therapy (cART) and those not treated with cART, compared with healthy controls. However, many studies included in that review did not take into account confounding factors such as body mass index (BMI), diabetes, hepatitis B virus (HBV) and hepatitis C virus (HCV) co-infections, or lifestyle factors [9,10,11,12]. Without considering these factors, it is impossible to conclusively determine HIV’s impact on epigenetic ageing. Furthermore, there are few studies with integrase inhibitor-based cART, a class of antiretroviral drugs that currently predominates [8,13].

In our study, global and site-specific DNA methylation was compared between cisgender men living with HIV successfully treated with integrase inhibitor-based cART and a control group of seronegative individuals. The site-specific markers, which have been selected based on previously published data, have been shown to be top differentially methylated regions in PLWH compared with controls or in terms of general methylation status correlated with ageing [14,15]; we have chosen NPTX2, FOXG1, and DPP6, which are primarily involved in neuronal development and synaptic function, and CNOT2, which is a core regulator of gene expression through its role in mRNA stability and transcriptional control. In all study participants, we also examined levels of DNA methyltransferase gene expression, which play an important role in DNA methylation [16]. Our main goal was to exclude confounding factors and ascertain if successfully treated HIV infection itself is an independent factor for DNA hypomethylation and accelerating epigenetic ageing.

## 2. Materials and Methods

### 2.1. Study Participants

This study included 98 people, who were divided into the study group and the control group. The study group (*n* = 48) included cisgender men living with HIV and receiving cART in Wroclaw, Poland. At the time of enrolment, participants had to be 24–40 years of age and have been receiving ART for at least two years. Successful integrase inhibitor-based cART was defined as HIV viral load < 40 copies per millilitre and a CD4+ lymphocyte count of at least 330 cells per microlitre at the time of enrolment. The control group (HIV−; *n* = 50) included cisgender male volunteers, also 24–40 years of age, with a negative fourth-generation HIV antigen/antibody test on enrolment. The relatively young age of participants (24–40 in both groups) was chosen to mitigate impact of undiagnosed disorders such as cardiovascular, metabolic or neoplastic disorders which might accelerate epigenetic ageing [7].

Exclusion criteria were: active neoplastic disease, clinically significant cardiovascular disorders, congenital metabolic disorders, active autoimmune diseases, diabetes, active infections other than HIV (including HBV infection with detectable HBs antigen, HCV replication, and untreated or inadequately treated syphilis), alcohol abuse, and current intravenous drug use. Among PLWH, people with current AIDS-defining disease (according to the definition of the Centers for Disease Control and Prevention [17]) were also excluded from trial participation.

Recruitment began in June 2023 and was completed in January 2024. A total of 49 people were included in the PLWH group and 50 in the control. All participants consented to participate in the study. All individuals filled out a questionnaire focusing on lifestyle factors that could potentially influence epigenetic ageing (height, weight, smoking, substance use, physical activity, diet and psychological stress). The questionnaire can be found in the Supplementary Materials.

Venous blood was collected from all participants in both groups for further testing, including assays for epigenetic markers of ageing. All participants underwent basic blood tests: complete blood count, lipid profiles including total cholesterol, high-density lipoprotein (HDL), low-density lipoprotein (LDL) and triglyceride levels, and creatinine, glucose and C-reactive protein (CRP) levels. In order to identify individuals meeting the exclusion criteria, the control group had fourth-generation HIV, HBs antigen, total anti-HCV antibody, and nontreponemal syphilis tests performed. These tests were also done in the PLWH group if the results were missing. PLWH had current HIV viral load and CD3+, CD4+ and CD8+ lymphocyte count measurements conducted. One person from the PLWH group was excluded from the study due to short time on cART and viral load exceeding 40 copies per millilitre. Ultimately, 48 individuals were included in the PLWH group and 50 individuals in the control group.

### 2.2. Epigenetic Analyses

DNA was isolated directly from the whole venous blood collected into EDTA-coated vials. An amount of 200 µL was used for DNA extraction using a commercial column spin method kit, the QIAamp DNA Mini Kit (QIAGEN, Hilden, Germany, cat. no. 51104) following the protocol for whole blood preparation. Whole blood was digested with proteinase K in lysis buffer; next, nucleic acids were precipitated with 96% ethanol and bound with the silica membrane-based column, washed with washing buffers, and finally eluted using 100 µL of elution buffer. Total RNA isolation from the whole blood was performed with the Monarch Spin RNA Isolation Kit (New England Biolabs, Ipswich, MA, USA, cat. No. T2110S) according to the protocol dedicated for whole blood. An amount of 200 µL of whole blood was mixed with 400 µL of stabilizing buffer and proteinase K and incubated at room temperature for 30 min. Next, RNA was precipitated using isopropanol and samples were placed on a silica membrane column, and RNA was bound, washed and eluted with 50 µL of nuclease-free water. The DNA and RNA purity and quantity were measured with Thermo Scientific NanoDrop (Thermo Fisher Scientific, Waltham, MA, USA).

Reverse transcription was performed with a High-Capacity cDNA Reverse Transcription Kit (Thermo Fisher Scientific, Waltham, MA, USA, cat. no 4368814). An amount of 500 ng of RNA was used for cDNA synthesis. Quantitative real-time PCR was carried out with Fast SYBR Green Master Mix (Thermo Fisher Scientific, cat. no 4385616) to evaluate gene expression (*DNMT1*, *DNMT3a*, *DNMT3b*). Primer sequences were manually designed and their amplification efficiency was validated with a standard curve. The specific primer sequences used have been previously described in Malodobra et al. [18]. Gene expression data were normalized to *β-actin* as the reference gene, and relative quantification was performed using the ∆∆Ct algorithm. Expression levels in the PLWH group were compared with the controls (the mean value of ΔΔCt of the control group was used as a calibrator factor).

DNA methylation assessment was conducted using the MagMeDIP qPCR Kit (Diagenode, Denville, NJ, USA, cat. no C02010021). An amount of 1200 ng of DNA was fragmented using Bioruptor Plus (Diagenode, Denville, NJ. USA). The protocol-defined 10 cycles of 45 s ON, 90 s OFF were applied. Next, 10% of the input was saved and DNA was precipitated using meC antibody (Diagenode) at 4 °C overnight. After DNA precipitation and extraction, its concentration (together with the input sample) was determined using the Pico488 dsDNA Quantification Reagent (Lumiprobe, Hanover, Germany, cat. no NC1878927). To obtain a calibration curve, human genomic DNA previously quantified using the Quantifiler™ Duo DNA Quantification Kit (Thermo Fisher Scientific, cat. no. 4387746) was used. Global DNA methylation was assessed based on the proportion of DNA immunoprecipitated with 5-methylcytosine antibodies (meC, Diagenode) relative to the total amount of input DNA, secured prior to precipitation. Analysis of methylation at selected genomic sites (CNOT2, DPP6, FOXG1 and NPTX2 methylation) was performed by real-time quantitative PCR (qPCR) using the Fast SYBR Green Master Mix reagent (ThermoFisher Scientific) and quantified according to the manufacturer’s protocol using the formula shown below. The primer sequences used for DNA methylation analysis are presented in Table S1 in the Supplementary Materials.

% recovery = 2^(CtIN − 3.32 − CtIP) × 100

Ct—Number of cycles, where the amplification curve crosses the threshold;
CtIN—Ct value of the 10% input sample—total DNA;
CtIP—Ct value of immunoprecipitated samples—methylated DNA;
3.32—Dilution factor, taking into account that input stands for 10%.

### 2.3. Statistical and Machine Learning Analysis

Continuous variables of the studied population were summarized using means, medians, standard deviations and interquartile ranges, while ordinal and binary variables were described using percentages, reported separately for both groups. The normality of each variable’s distribution was assessed using the Shapiro–Wilk test. To evaluate differences in variable distributions between groups, Welch’s test was applied for variables that could not be rejected as non-normal in both groups, accommodating possibly unequal variances. For non-normally distributed variables, the two-sided Mann–Whitney U test was used as a nonparametric alternative.

Associations between variables were explored using Spearman rank correlation, computed independently for each group. Three levels of significance were indicated: the conventional threshold of 0.05, along with 0.01 and 0.001. For the primary analysis, a significance cutoff of 0.01 was chosen—more stringent than the standard 0.05.

The objective of this analysis was to determine whether HIV infection (iv) significantly impacts the dependent variables (dvs)—epigenetic ageing markers (global DNA methylation; CNOT2, DPP6, FOXG1 and NPTX2 methylation; *DNMT1*, *DNMT3a* and *DNMT3b* expression levels)—while minimizing bias introduced by covariates. For each dependent variable, the null hypothesis was that HIV infection has no effect; under the alternative, models incorporating covariates plus HIV infection would demonstrate substantially superior predictive performance (e.g., higher R^2^) compared to models using covariates alone. Several challenges complicated this evaluation: (1) non-normal distributions and heterogeneous variances among the dependent variables; (2) substantially different distributions of some key covariates between the HIV+ and HIV- groups; (3) collinearity among covariates; (4) potentially nonlinear relationships between variables; and (5) a limited number of observations (n = 98) relative to the number of covariates (32). A fully nonparametric framework was adopted to circumvent limitations of standard MANCOVA assumptions (which cannot be used here). Challenges (1) and (2) were addressed through permutation tests using R^2^ as the test statistic, with Holm–Bonferroni adjustment for multiple testing across dependent variables. Challenges (3) and (4) were mitigated by employing robust, ensemble-based tree regressors [19], the Random Forest Regressor, Extra Trees Regressor, and Gradient Boosting Regressor, which inherently handle collinearity and nonlinearity without parametric assumptions. The Linear Regressor was also added as a reference. To counter challenge (5) and reduce overfitting risk in this low-n setting, leave-one-out cross-validation was used. In implementation, each regressor was fitted separately for every dependent variable, first using only covariates (baseline model) and then adding HIV status (full model), with leave-one-out cross-validation to compute out-of-sample R^2^. The significance of HIV infection was evaluated via permutation tests (10,000 iterations per model), generating a null distribution of ΔR^2^ (full minus baseline) by randomly shuffling HIV status labels.

Empirical *p*-values were derived from the proportion of permuted ΔR^2^ exceeding the observed value.

## 3. Results

Table 1 and Table 2 show the characteristics of the study population. Participants were all males, aged 24–40 years, with significantly higher mean age in the PLWH group (34 vs. 29.8; *p* < 0.001). Out of the dependent variables, only *DNMT3a* expression was distributed similarly in both groups (*p* = 0.114). Considering covariates, most were similarly distributed between the two groups (*p* ≥ 0.05). The most notable difference was in smoking habits, with all smoking-related covariates significantly higher in the PLWH group than in the HIV- group (*p* < 0.001 for smoking status, years smoked, cigarettes per day, and pack-years). Other covariates with different distributions were: haemoglobin (mean in the PLWH group 15.227 g/dL; in the HIV- group 14.904 g/dL; *p* < 0.05) and creatinine (mean in the PLWH group 0.955 mg/dL; in the HIV- group 0.916 mg/dL; *p* < 0.05).

**Figure 1:**
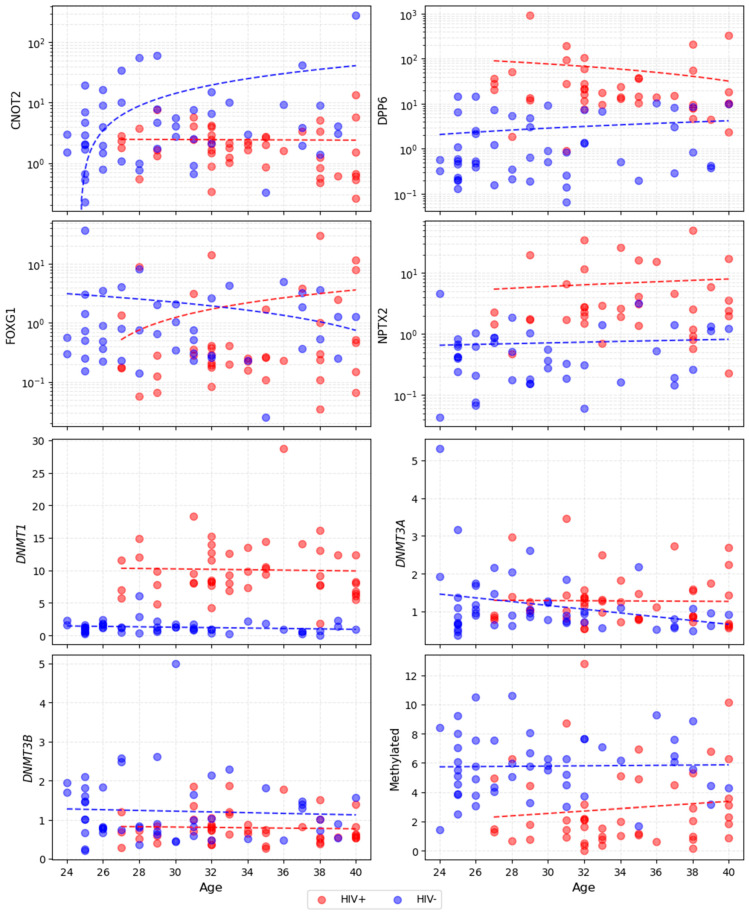
Relation between dependent variables and chronological age. Dashed lines are fitted by a least-squared Linear Regressor and are for illustrative purposes only (no statistically significant correlations were observed). Note that OY is in a logarithmic scale for CNOT2, DPP6, FOXG1 and NPTX2.

Correlations in the HIV- group and in the PLWH group are presented in Figures S1 and S2 in the Supplementary Materials. Strong correlations were observed consistently across both groups, forming clear clusters: BMI/weight/height; smoking-related variables; CNOT2/DPP6/FOXG1 methylation levels and global DNA methylation; and CD3+/CD4+/CD8+ lymphocyte counts for PLWH (these parameters were not measured for the control group). Total cholesterol-LDL and total cholesterol/triglyceride correlations were present in both groups but varied in strength. Weight correlations shifted between groups, linking more to HDL/triglycerides/CRP in controls and to LDL/total cholesterol in PLWH. Smoking variables were linked to other covariates (e.g., fruit/vegetables, FOXG1 methylation) only among PLWH. Notably, age showed almost no significant correlations with epigenetic variables in either group (all *p* ≥ 0.05; see also Figure 1).

The results of machine learning analysis of the relationship between HIV infection and epigenetic markers are shown in Table 3. The LR model was used as a reference and performed worse than the other regressors, especially for CNOT2, DPP6 and FOXG1 methylation levels, due to the outlier sensitivity. It will not be discussed in further analysis. The models exhibited poor baseline fits for CNOT2, DPP6, FOXG1 and NPTX2 methylation levels, as well as *DNMT3a* and *DNMT3b* expression levels across regressors, with R^2^ values remaining negative, indicating limited ability to explain variance using covariates alone. This may stem from overfitting complex tree-based models to a small dataset (n = 98) or suboptimal hyperparameter tuning.

HIV status (independent variable) had no observable impact on predicting CNOT2, FOXG1 methylation, and *DNMT3a* expression (adjusted *p* = 1.0 across all nonlinear regressors). Similarly, for DPP6 methylation and *DNMT3b* expression no regressor yielded a significant adjusted improvement; there were some unadjusted significant results (DPP6, Extra Trees, *p* = 0.016 and *DNMT3b*, Extra Trees, *p* = 0.016; Gradient Boosting, *p* = 0.012).

In contrast, a strong association was evident for *DNMT1* expression (*p* < 0.001 unadjusted and adjusted; R^2^ > 0.5 in reference models for all nonlinear regressors, with ΔR^2^ > 0.513).

Similarly, HIV status significantly influenced global DNA methylation level (adjusted *p* ≤ 0.028; ΔR^2^ ≈ 0.115–0.183 despite baseline R^2^ ≈ 0).

For NPTX2 methylation, the evidence was inconclusive: Extra Trees and Gradient Boosting supported a relation (adjusted *p* ≤ 0.002; ΔR^2^ = 0.212 and ΔR^2^ = 0.176), while Random Forest rejected it (adjusted *p* = 0.357; negligible ΔR^2^). Given the poor overall fit, this potential association may be attributable to random variation.

## 4. Discussion

The results of our study confirm HIV infection’s impact on global DNA hypomethylation and thus the acceleration of epigenetic ageing, which has already been described in many studies [8,12,20,21]. In our trial, the average level of global DNA methylation was significantly lower among PLWH than in the HIV- control group. It is important to note the significantly higher age among PLWH in our study compared to the HIV- controls. However, in contrast to the previous studies, we have showed almost no (linear) correlation between the tested epigenetic markers, including global DNA methylation and DNA methyltransferase gene expression levels, and chronological age in either group. This surprising observation can be linked to the similar, relatively young age of all study participants (between 24 and 40) and the fact that epigenetic age is only partially related to chronological age. A similar observation in relation to this age group (in contrast to older participants) has been found in at least one previous study on related issues [22].

We have also analysed the relationship between HIV infection and levels of methyltransferase expression (*DNMT1*, *DNMT3a*, *DNMT3b*), which plays a key role in DNA methylation [16,23]. The average level of *DNMT1* expression was almost eight times higher among PLWH than in the control group. This is consistent with previously published data indicating that HIV induces *DNMT1* expression in a non-specific tissue manner and affects DNA hypomethylation [23]. It was proposed that HIV infection stimulates *DNMT1* expression via expression of the early HIV genes, especially *Nef*, that induce *DNMT1* promoter activity [24]. Several studies published to date have also found an association between HIV infection and higher levels of *DNMT3a* and *DNMT3b* expression [23,25]. In our study, we did not find such a correlation: the level of *DNMT3a* expression was slightly higher in the PLWH group, but the difference was not statistically significant, while the level of *DNMT3b* expression was actually lower among PLWH than in the controls. Of note, *DNMT1* is responsible for maintaining methylation patterns following DNA replication [26], while *DNMT3a* and *DNMT3b* regulate de novo methylation [27]. Perhaps, having been treated for longer periods (at least two years on cART), the PLWH in our group may have contributed to the lack of increase in the expression of *DNMT3a* and *DNMT3b*. A pattern of *DNMT1* expression and global DNA methylation similar to that observed in our study was reported in obese mice fed a high-fat diet [28]. We believe that the occurrence of global DNA hypomethylation despite increased *DNMT1* expression in PLWH may be potentially related to several previously described mechanisms. These include impaired DNMT function, resulting in reduced enzyme activity as well as impaired recruitment of the cofactor (UHRF1), which is essential for the DNA methylation process [29]. It is also possible that increased *DNMT1* expression represents a compensatory response to active DNA demethylation [30] or that it is related to the above-mentioned induction of *DNMT1* promoter activity by viral protein [24]. However, further research is required to validate these mechanisms.

Models developed based on machine learning allow for strong prediction of global DNA methylation and *DNMT1* expression levels among the considered regression models, and indicate a strong relationship. They confirmed a strong association between HIV infection and global DNA hypomethylation and increased *DNMT1* expression reported in previous studies [8,23]. No significant association was found between HIV infection and CNOT2, DPP6, FOXG1 and NPTX2 methylation levels or *DNMT3a* and *DNMT3b* expression levels. To the best of our knowledge, this is the first study using advanced machine learning methods to confirm or exclude an association between HIV infection and epigenetic ageing markers. These methods enable robust detection of potentially nonlinear relationships between HIV status and epigenetic markers while simultaneously controlling for multiple covariates, including chronological age and lifestyle factors.

It is worth noting that site-specific DNA methylation was decreased at only two of the four analysed loci (CNOT2 and FOXG1) in PLWH patients compared to the control group, while the other two loci (DPP6 and NPTX2) were hypermethylated (however, as stated above, after taking into account confounding factors, these differences between PLWH and the control group were not significant). Although global DNA methylation generally decreases with age (while certain site-specific methylation loci become hypermethylated), numerous loci may display distinct methylation patterns in relation to ageing. Previously published data indicate that approximately 60.5% of methylation sites undergo age-associated decreases in methylation, whereas the remaining 39.5% exhibit increased methylation [31]. Finally, Kenneth Day et al. showed that site-specific DNA methylation changes during ageing are tissue-specific and may result from tissue-specific gene expression. They further concluded that age-associated changes in DNA methylation are multidirectional and driven by multiple factors [32]; therefore, a simple model is insufficient to fully explain the epigenetic alterations that occur during the ageing process. The above considerations may at least partially explain the divergent patterns of site-specific DNA methylation in our study.

Among the mechanisms responsible for the influence of HIV infection on epigenetic ageing, the most frequently mentioned are accelerated immunosenescence, chronic inflammation, and ART side effects [4,8,33,34,35,36,37]. However, Sehgal et al. also indicate that antiretroviral treatment might be particularly effective in reversing the epigenetic ageing process [38]. To date, very few studies have been published on epigenetic ageing in PLWH treated with integrase inhibitors, which are currently the most important class of antiretroviral drugs [8,13]. It appears that at least some older-generation antiretroviral drugs may have contributed significantly to the acceleration of epigenetic ageing in PLWH by causing side effects that are not observed during treatment with integrase inhibitors [4]. However, the results of our study indicate that even in PLWH effectively treated with integrase inhibitors, global DNA methylation is significantly reduced compared to uninfected individuals, indicating accelerated epigenetic ageing.

The main limitations of our study are the relatively small size of the studied population and that the group is limited only to men. Age at HIV diagnosis, timeline of diagnosis and immune status at diagnosis were not included in the analyses due to missing data in some participants’ medical history, especially if they were diagnosed in the very distant past. Another limitation is the significant difference in average age between the study and control groups, which was, however, taken into account in machine learning analyses.

In summary, the results indicate that in PLWH on effective integrase inhibitor-based cART, HIV infection remains an independent factor causing global DNA hypomethylation and thus accelerating epigenetic ageing. It is also significantly associated with higher expression of the *DNMT1* methyltransferase gene. However, we did not find any significant association with CNOT2, DPP6, FOXG1 and NPTX2 methylation levels or *DNMT3a* and *DNMT3b* expression levels. We believe that our study, which takes into account a very wide range of confounding factors, can serve as a starting point for further similar studies involving larger groups, which will also take into account other variables that we were unable to consider in the present study.

## Figures and Tables

**Table 1:** Characteristics of the study participants: ordinal and binary variables.

Variable	Range	Study Participants, %										*p*-Value
		PLWH (N = 48)					HIV− (N = 50)					
		0	1	2	3	4	0	1	2	3	4	
Alcohol drinking frequency	0–3 *	10	44	44	2	-	22	36	42	-	-	0.331
Fast food eating freq.	1–4 **	-	52	42	4	2	-	46	48	4	2	0.679
Fatty food eating freq.	1–4 **	-	11	46	22	22	-	10	36	38	16	0.653
Intravenous drug use	0–2 ***	96	4	0	-	-	100	0	0	-	-	0.151
Inhaling drugs	0–2 ***	69	21	10	-	-	82	12	6	-	-	0.134
Physical activity freq.	1–4 **	-	15	35	23	27	-	14	34	22	30	0.798
Coping with stress	1–4 ****	-	5	23	43	30	-	2	20	44	32	0.654
Stress level	0–4 *****	38	2	23	19	12	34	12	22	24	6	0.583
Fruit and vegetable eating freq.	1–4 **	-	0	7	11	80	-	2	28	12	58	0.013
HIV RNA	0–1 ******	33	67	-	-	-	100	0	-	-	-	<0.001
Smoking	0–2 ***	29	27	44	-	-	72	22	6	-	-	<0.001

* 0, never; 1, once a month; 2, once a week; 3, daily; ** 1, never; 4, regularly; *** 0, never; 1, in the past; 2, currently; **** 1, not well; 4, well; ***** 1, low; 4, very high; ****** 0, not detected; 1 means < 40 copies/mL. *p*-values were calculated using the two-sided Mann–Whitney U test. Variables are grouped by whether they are similarly distributed (*p* ≥ 0.05) or differently distributed (*p* < 0.05), with similarly distributed variables listed first, and sorted alphabetically within each group.

**Table 2:** Characteristics of the study participants: continuous variables.

Variable	Study Participants						Similarity	
	HIV+ (N = 48)			HIV− (N = 50)				
	Mean (Median)	SD (IQR)	NPv	Mean (Median)	SD (IQR)	NPv	Test	*p*-Value
Age [years]	33.96 (33)	4.021 (6.250)	0.010	29.76 (29)	4.732 (6)	<0.001	U	<0.0001
Height [cm]	180.0 (180)	6.738 (9)	0.406 *	181.2 (180)	7.280 (10)	0.689 *	T	0.375 *
Weight [kg]	79.81 (80)	13.73 (17.75)	0.548 *	80.67 (77.50)	15.28 (19)	0.009	U	0.972 *
BMI [kg/m^2^]	24.60 (23.70)	3.774 (3.935)	0.460 *	24.47 (23.73)	3.836 (4.643)	0.001	U	0.582 *
HGB [g/dL]	15.23 (15.50)	1.120 (1.325)	0.036	14.90 (15)	0.870 (1.175)	0.177 *	U	0.036
WBC [k/µL]	6.300 (6.195)	1.483 (2.263)	0.710 *	6.981 (6.690)	1.479 (1.993)	0.008	U	0.051 *
PLT [k/µL]	241.3 (239.5)	54.49 (57.75)	0.073 *	247.2 (238.5)	58.23 (67.50)	0.059 *	T	0.608 *
Creatinine [mg/dL]	0.955 (0.945)	0.139 (0.233)	0.368 *	0.916 (0.885)	0.147 (0.130)	<0.001	U	0.042
Glucose [mg/dL]	86.72 (86.50)	9.582 (10)	0.002	88.48 (84.50)	15.83 (11.50)	<0.001	U	0.790 *
Total cholesterol [mg/dL]	189.0 (188.9)	41.43 (50.05)	0.219 *	193.4 (195)	37.97 (52.50)	0.847 *	T	0.588 *
HDL [mg/dL]	49.97 (50)	10.71 (14.50)	0.931 *	49.64 (49)	8.461 (11.50)	0.378 *	T	0.868 *
LDL [mg/dL]	122.9 (117.5)	40.03 (47.40)	0.112 *	120.8 (122)	33.57 (49.50)	0.785 *	T	0.780 *
Triglycerides [mg/dL]	138.3 (122.5)	98.15 (73.32)	<0.001	153.8 (142.5)	87.52 (72.25)	<0.001	U	0.118 *
CRP [mg/L]	1.757 (1)	1.646 (0.810)	<0.001	2.316 (1)	4.301 (0.475)	<0.001	U	0.588 *
Years of smoking	6.295 (5)	6.522 (10)	<0.001	1.163 (0)	2.664 (1)	<0.001	U	<0.001
Cigarettes per day	7.064 (6.500)	6.947 (10)	<0.001	1.410 (0)	2.969 (0.066)	<0.001	U	<0.001
Pack-years	3.187 (1.375)	4.998 (3.812)	<0.001	0.301 (0)	0.867 (0.020)	<0.001	U	<0.001
Sleep hours per day	7.120 (7)	1.009 (1.500)	0.048	7.275 (7.500)	0.977 (1.500)	0.182 *	U	0.574 *
CD3 [cells/µL]	1599 (1523)	401.8 (619.5)	0.076 *	-	-	-	-	-
CD4 [cells/µL]	762.0 (722.5)	231.9 (233)	0.073 *	-	-	-	-	-
CD8 [cells/µL]	747.8 (713.5)	243.2 (380.8)	0.257 *	-	-	-	-	-
Methylated [% Input.]	2.874 (1.923)	2.778 (3.461)	<0.001	5.777 (5.781)	2.137 (3.128)	0.868 *	U	<0.001
CNOT2 [% Input.]	2.428 (1.818)	2.297 (1.888)	<0.001	13.55 (3.045)	41.10 (7.221)	<0.001	U	0.004
DPP6 [% Input.]	59.71 (16.36)	150.7 (26.71)	<0.001	2.845 (0.621)	3.950 (4.145)	<0.001	U	<0.001
FOXG1 [% Input.]	2.186 (0.267)	5.428 (0.599)	<0.001	2.210 (0.695)	5.677 (1.759)	<0.001	U	0.007
NPTX2 [% Input.]	6.830 (2.480)	10.59 (4.023)	<0.001	0.713 (0.420)	0.862 (0.696)	<0.001	U	<0.001
*DNMT1*	10.11 (9.200)	4.366 (4.746)	<0.001	1.277 (1.178)	0.946 (0.992)	<0.001	U	<0.001
*DNMT3A*	1.281 (1.141)	0.669 (0.633)	<0.001	1.174 (0.937)	0.837 (0.587)	<0.001	U	0.114 *
*DNMT3B*	0.794 (0.709)	0.398 (0.379)	<0.001	1.221 (0.947)	0.841 (0.956)	<0.001	U	0.005

Abbreviations: SD, standard deviation; NPv, normality test *p*-value. *p*-values were calculated using (U) two-sided Mann–Whitney U test or (T) Welch’s *t*-test. CD3, CD4 and CD8 lymphocytes were measured only within HIV+ group. Normality was tested using Shapiro–Wilk test. Results with *p* > 0.05 were significant and are marked with asterisk.

**Table 3:** Results of fitting regression models to predict dependent variables.

Variable	Model		R^2^		*p*-Value	
		Permuted	Baseline	Δ	Base	Adjusted
CNOT2	ET	−0.038	−0.071	−0.033	0.944	1.0
	GB	−0.086	−0.085	0.001	0.450	1.0
	LR	−0.864	−0.942	−0.078	0.814	1.0
	RF	−0.073	−0.074	−0.001	0.456	1.0
*DNMT3A*	ET	−0.148	−0.150	−0.002	0.435	1.0
	GB	−0.396	−0.371	0.025	0.264	1.0
	LR	−0.555	−0.561	−0.006	0.185	1.0
	RF	−0.275	−0.266	0.009	0.418	1.0
*DNMT1*	ET	0.028	0.541	0.513	<0.001 *	<0.001 *
	GB	−0.071	0.545	0.616	<0.001 *	<0.001 *
	LR	−0.147	0.342	0.489	<0.001 *	<0.001 *
	RF	0.029	0.593	0.564	<0.001 *	<0.001 *
*DNMT3B*	ET	−0.297	−0.162	0.135	0.016 *	0.128
	GB	−0.731	−0.603	0.128	0.012 *	0.095
	LR	−0.504	−0.466	0.038	0.051	0.411
	RF	−0.380	−0.346	0.034	0.201	1.0
DPP6	ET	−0.125	−0.067	0.058	0.014 *	0.114
	GB	−0.287	−0.228	0.059	0.407	1.0
	LR	−1.039	−0.983	0.056	0.08	0.636
	RF	−0.099	−0.076	0.023	0.196	1.0
FOXG1	ET	−0.302	−0.317	−0.015	0.651	1.0
	GB	−0.549	−0.613	−0.064	0.773	1.0
	LR	−1.287	−1.347	−0.06	0.408	1.0
	RF	−0.208	−0.204	0.004	0.468	1.0
Global methylation	ET	−0.044	0.076	0.12	0.003 *	0.026 *
	GB	−0.255	−0.072	0.183	0.004 *	0.028 *
	LR	−0.423	−0.297	0.126	0.014 *	0.108
	RF	−0.019	0.097	0.116	<0.001 *	0.002 *
NPTX2	ET	−0.273	−0.061	0.212	<0.001 *	<0.001 *
	GB	−0.446	−0.270	0.176	<0.001 *	0.002 *
	LR	−0.683	−0.540	0.143	0.025 *	0.201
	RF	−0.248	−0.199	0.049	0.045 *	0.357

Abbreviations: ET, Extra Trees; GB, Gradient Boosting; LR, Linear Regression; RF, Random Forest. Quality of regression is given by R^2^ score. Permuted R^2^ is mean over permuted iv, baseline is mean over data with true iv, Δ is difference between them. Results with *p* < 0.05 were significant (without adjustment, col. Base; with adjustment, col. Adjusted) and are marked with asterisk.

## Data Availability

The original contributions presented in this study are included in the article/Supplementary Material. Further inquiries can be directed to the corresponding author.

## Supplementary Materials

The following supporting information can be downloaded at https://www.mdpi.com/article/10.3390/v18020199/s1: Part S1: Questionnaire for study participants. Part S2: Primer sequences used for DNA methylation analysis. Part S3: Supplementary figures. Part S4: Spreadsheet containing research data.
