# Supplementary material for: HIV Infection as an Independent Factor Accelerating Epigenetic Ageing in Men Treated with Integrase Inhibitors: A Case–Control Study
Source: Viruses. 2026 Feb 2;18(2):199. doi: 10.3390/v18020199 (PMC12945144; doi:10.3390/v18020199)
Supplement: Supplementary file 1 [file viruses-18-00199-s001.zip › HIV case control Supplementary Materials S1.pdf]

HIV infection as an independent factor accelerating epigenetic ageing in men treated with integrase inhibitors: a case-control study

Supplementary Materials

Part 1: Questionnaire for study participants

1. How old are you? …………….

2. How tall are you? …………….. How much do you weigh? ……………………

3. Do you smoke cigarettes (or other tobacco products)?

YES / NO, BUT I SMOKED IN THE PAST / I HAVE NEVER SMOKED

4. If you currently smoke cigarettes or have smoked in the past, please answer:

How many years have you been smoking (smoked)? …………

How many cigarettes do you smoke (smoked) on average per day? ………………

5. How often do you drink alcohol? Please select the answer that is closest to the truth:

EVERY DAY / ONCE A WEEK / ONCE A MONTH / I DO NOT DRINK AT ALL

What type of alcohol and in what quantities? ……………………………………………

6. Do you take intravenous drugs?

YES / NO, BUT I HAVE TAKEN THEM IN THE PAST /

I HAVE NEVER TAKEN THEM

7. Do you take inhaled drugs?

YES / NO, BUT I HAVE IN THE PAST /

I HAVE NEVER

8. How often do you do sports or other physical activities?

Please rate on a scale from 1 (never) to 4 (regularly): ………………………..

Please specify the type of sport and duration of exercise:
……………………………………………………………………………………..…………………………
……………………………………

9. Please rate on a scale of 1 (never) to 4 (many times):

How often have you eaten fruit and vegetables in the last 7 days? …………………..

How often have you eaten fatty foods in the last 7 days? ……………………

How often have you eaten fast food in the last 7 days? …………………….

10. How many hours do you sleep on average each day? ………………………………

11. In your opinion, are you chronically or regularly exposed to stress? YES / NO

If so, how severe is the stress?

Please rate on a scale from 1 (mild) to 4 (very severe): …………………………

12. In your opinion, how well do you cope with excessive stress (pressure)?

Please rate on a scale from 1 (I do not cope well) to 4 (I cope well): ………………
