# Supplementary material for: HIV Infection as an Independent Factor Accelerating Epigenetic Ageing in Men Treated with Integrase Inhibitors: A Case–Control Study
Source: Viruses. 2026 Feb 2;18(2):199. doi: 10.3390/v18020199 (PMC12945144; doi:10.3390/v18020199)
Supplement: Supplementary file 1 [file viruses-18-00199-s001.zip › HIV case control Supplementary Materials S2.pdf]

HIV infection as an independent factor accelerating epigenetic ageing in men treated with integrase inhibitors: a case-control study

Supplementary Materials

Part 2

Table S1. Primers sequences used for DNA methylation analysis.

Gene	Primer	Sequence (5'→3')
CNOT2	forward	AGTGAGGAGGGGAGGAGTG
	reverse	AGAAGGCTCTGGCCCTGAG
DPP6	forward	AGAGAAAGCACAGCCAGAG
	reverse	AGCAGCAGCAGCAAGGCAAG
FOXG1	forward	CTTTTGCTACATGACTTGCC
	reverse	ATCTCCCATGTCCAGCATCAC
NPTX2	forward	ACTTTCTCCTCAGCCTGTG
	reverse	CTCTGCCTCAGGAAAGGAG
