# Supplementary figures and images for: HIV Infection as an Independent Factor Accelerating Epigenetic Ageing in Men Treated with Integrase Inhibitors: A Case–Control Study
Source: Viruses. 2026 Feb 2;18(2):199. doi: 10.3390/v18020199 (PMC12945144; doi:10.3390/v18020199)

## Supplementary Materials

## Part 3: SUPPLEMENTARY FIGURES

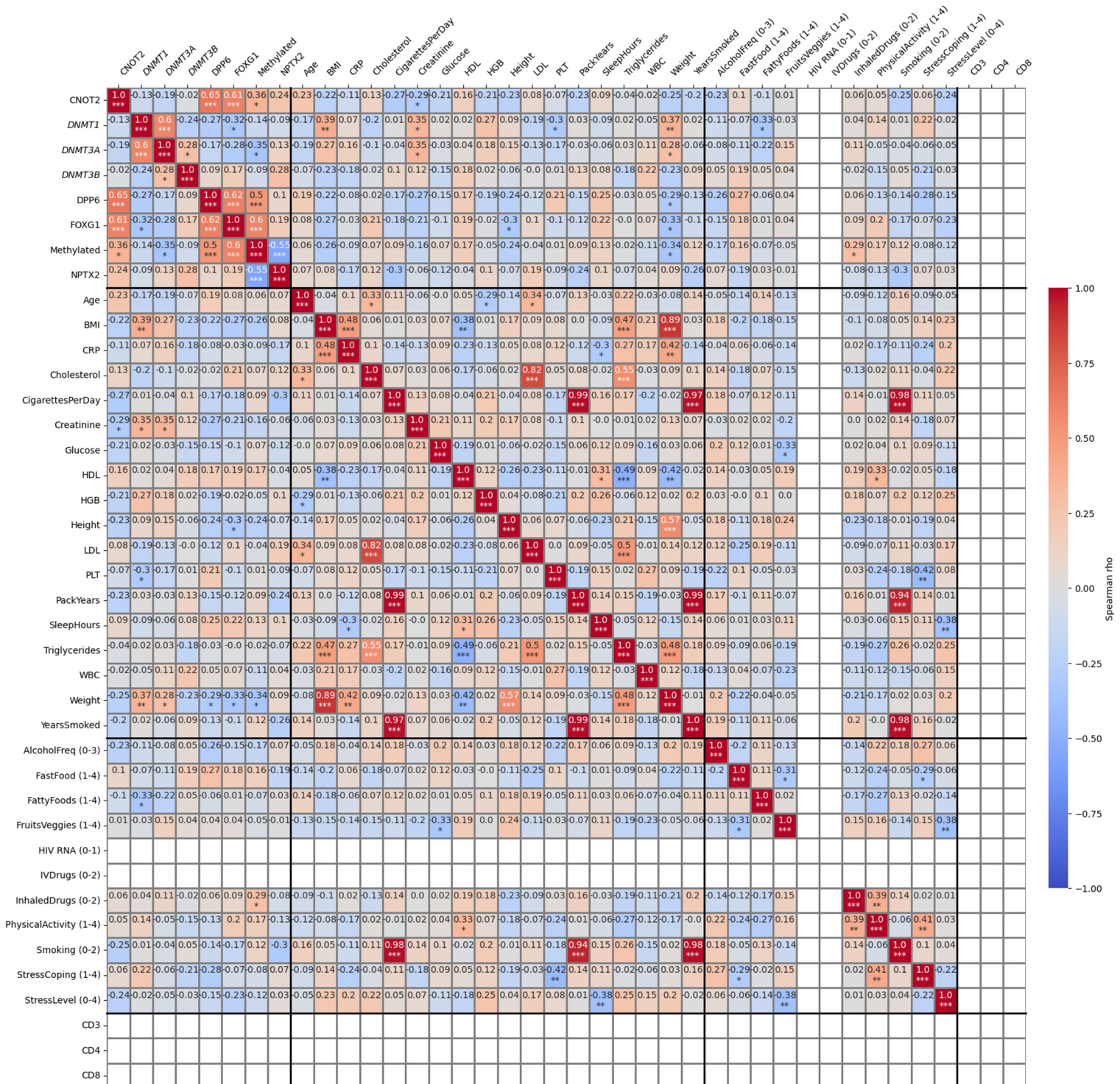

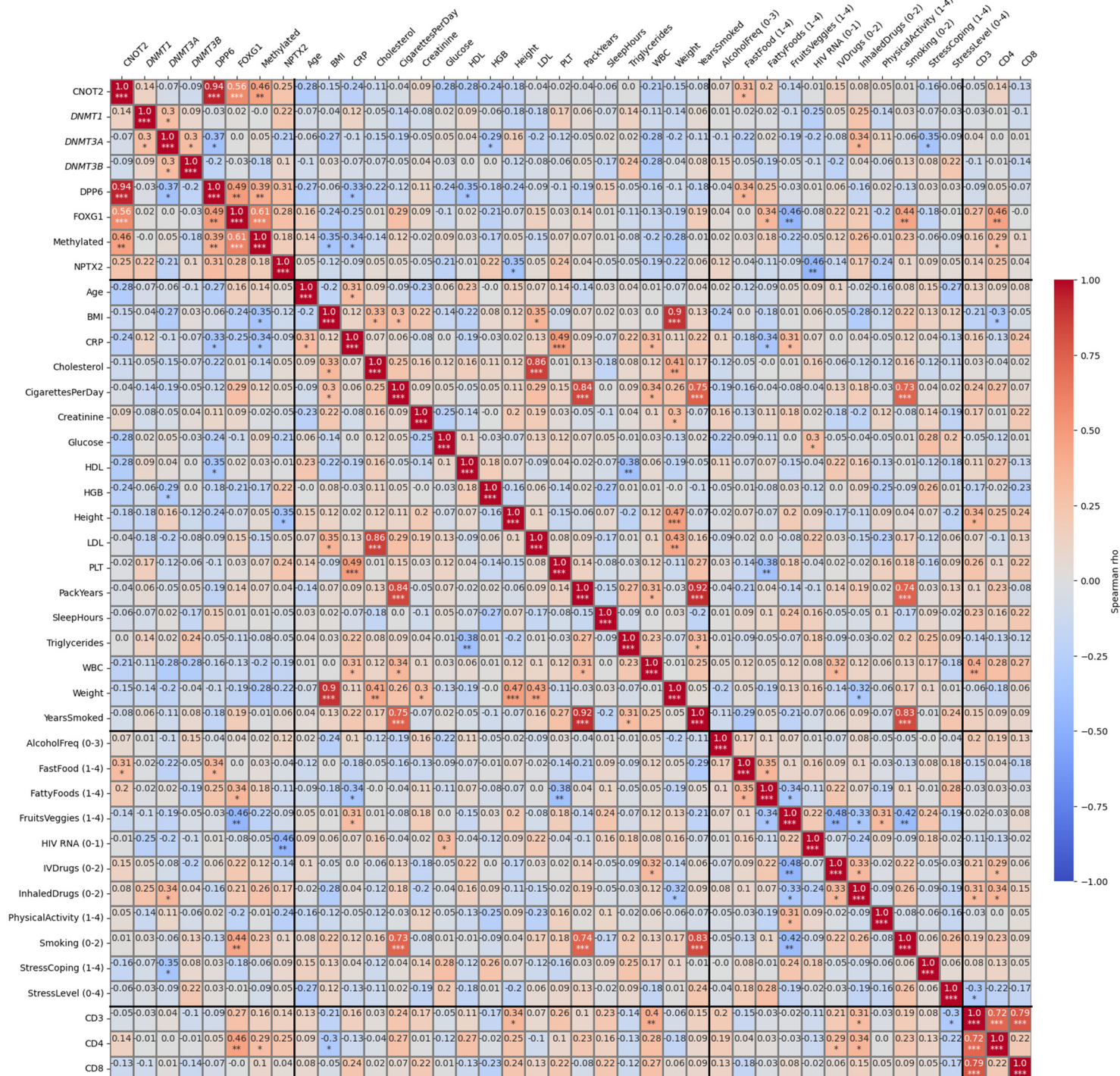

Supplement: Supplementary file 1 [file viruses-18-00199-s001.zip › HIV case control Supplementary Materials S3.pdf]
